# Psycho-emotional stress, folliculogenesis, and reproductive technologies: clinical and experimental data

**DOI:** 10.18699/VJGB-22-53

**Published:** 2022-08

**Authors:** A.L. Levinson, T.N. Igonina, I.N. Rozhkova, E.Yu. Brusentsev, S.Ya. Amstislavsky

**Affiliations:** Novosibirsk Center of Reproductive Medicine, Novosibirsk, Russia Novosibirsk State University, Novosibirsk, Russia; Institute of Cytology and Genetics of the Siberian Branch of the Russian Academy of Sciences, Novosibirsk, Russia; Institute of Cytology and Genetics of the Siberian Branch of the Russian Academy of Sciences, Novosibirsk, Russia; Institute of Cytology and Genetics of the Siberian Branch of the Russian Academy of Sciences, Novosibirsk, Russia; Institute of Cytology and Genetics of the Siberian Branch of the Russian Academy of Sciences, Novosibirsk, Russia Novosibirsk State University, Novosibirsk, Russia

**Keywords:** stress, long-term effects, folliculogenesis, assisted reproductive technologies, preimplantation embryo, стресс, отдаленные эффекты, фолликулогенез, вспомогательные репродуктивные технологии, преимплантационный эмбрион

## Abstract

Modern life, especially in large cities, exposes people to a high level of noise, high density of population, disrupted sleeping, large amount of excessive and controversial information as well as to other negative factors; all this may cause chronic psycho-emotional stress. The latest publications often use the term “Syndrome of megalopolis”, which means disruption of sleeping, high anxiety, and altered reproductive function. Medical treatment of infertility may also be considered as a stress factor, especially when infertility lasts for years and is aggravated with emotional frustration. Long-lasting distress may worsen health in general and suppress reproductive function, in particular. The review presents the data on the effects of maternal stress on folliculogenesis, especially when assisted reproductive technologies (ARTs) are used. Clinical data are presented alongside data from laboratory animal experiments. Different maternal stress models are taken into account in respect of their inf luence on oocyte maturation and embryo development. The interfering of psycho-emotional stress and reproductive function is the focus of the review. In these situations, exogenous hormones compensate for the stress-related disruption of the hypothalamic-pituitary-gonadal axis. When ARTs are implemented, stress-induced disruption of oogenesis is realized not via a decrease in hypothalamic and pituitary hormones, but by other ways, which involve paracrine mechanisms described in this review. Based on the literature analysis, one may conclude that stress negatively affects oocyte maturation in the ovary and suppresses subsequent embryo development. The role of some ovarian paracrine factors, such as BDNF, GDF-9, HB-EGF, TNF-α, and some others has been elucidated

## Abstract (ru)

Темп современной жизни, уровень шума, плотность населения, особенно в больших городах, нарушение ритма сна и отдыха, количество получаемой и обрабатываемой разнонаправленной информации и другие факторы современной жизни в мегаполисах могут приводить к хроническому психоэмоциональному стрессу. Все чаще в публикациях последних лет появляется понятие «синдром мегаполиса», объединяющее нарушения сна, проявления тревожности и изменение репродуктивной функции. Медицинские процедуры также считаются стрессовыми факторами; более того, само состояние бесплодия, как и его лечение, нередко является затяжным, длится годами, что в свою очередь способствует субадаптивным и неадаптивным реакциям организма, усугубляющим проблему. Пребывание в состоянии дистресса может отражаться на состоянии здоровья в целом и на реализации репродуктивной функции в частности. В обзоре обсуждаются литературные сведения, касающиеся влияния стресса матери на фолликулогенез, в том числе на фоне применения вспомогательных репродуктивных технологий (ВРТ). Сопоставлены клинические данные и результаты экспериментальных исследований на лабораторных животных о влиянии психоэмоционального стресса до и во время беременности на созревание ооцитов и развитие эмбрионов. Особое внимание уделено влиянию психоэмоционального стресса на репродуктивную функцию на фоне применения ВРТ, когда введение экзо- генных гормонов компенсирует вызванное стрессом нарушение функции гипоталамо-гипофизарно-гонадной оси. При применении ВРТ стресс-индуцированное нарушение оогенеза реализуется не через путь снижения выработки гормонов гипоталамуса и гипофиза, а через другие механизмы, в частности паракринные, которые описаны в этом обзоре. В результате проведенного анализа литературы можно сделать заключение о том, что стресс негативно влияет на развитие ооцитов яичника, а также последующее развитие полученных из них эмбрионов, выявлена роль некоторых паракринных факторов яичника, таких как BDNF, GDF-9, HB-EGF, TNF-α и др., которые задействованы в этих процессах.

## Introduction

Adult reproductive function and children’s health are in focus of scientific interest and of a great public concern. The implementation of assisted reproductive technologies (ARTs) into clinical practice helps to overcome many types of infertility, miscarriage, and to prevent monogenic diseases in children. At the same time, patients in ART clinics often report that the infertility itself as well as its treatment is the traumatic experience, which may lead to the anxiety and even depression (Cousineau, Domar, 2007; Rockliff et al., 2014). Thus, chronic psycho-emotional stress that affects both women and men during infertility treatment and implementation of the ART in particular, are significant factors affecting fertility.

There are numeric evidences of the negative impact of chronic stress on the human well-being, on the mammalian physiology in general, and on the reproductive function in particular (Louis et al., 2011; Muscatell, Eisenberger, 2012). The effects of maternal stress during pregnancy on the body weight of newborns and on the neurodevelopment in children are reported; moreover, there are evidences that prenatal stress affects the behavior and other phenotypic characteristics of different animals (Weinstock, 2008, 2016; Ragaeva et al., 2018; Fitzgerald et al., 2021).

Although the effects of psycho-emotional stress are described in the medical literature and on the laboratory animal models, these two areas of research are mostly developing independently. It should be noted, that nowadays ART is widely used in medical practice, thus the effects of psycho-emotional stress on reproduction, inclu- ding stress arising from the use of ART, as well as studying the mechanisms underlying these effects are of great concern. The objective of this article is to review and to systematize the accumulated experimental and clinical data describing the effects of chronic psycho-emotional stress on gametogenesis, fertility, ART outcomes, and the offspring health; to review animal models used in such experiments and to outline possible ways aiming to mitigate the adverse effects of stress associated with the use of ARTs. At the same time, experimental data obtained on animal models are compared with clinical observations published in the medical literature. The effects of stress on folliculogenesis and embryogenesis, as well as on the ART-born offspring, both in humans and in experimental animals, are analyzed.

## Modeling psycho-emotional stress in laboratory animals

In experimental studies aimed to elucidate the effects of chronic psycho-emotional stress, including the stress associated with the use of ARTs, on the development of oocytes and early embryos, animal model of restriction stress (Burkus et al., 2013; Gao et al., 2016), the predator exposure model (Liu et al., 2012; Di Natale et al., 2019), or the model of chronic unpredictable mild stress – CUMS (Wu L.M. et al., 2012a, b; Gao et al., 2016) are most frequently used. Plasma levels of corticosteroids, adrenocorticotropic hormone, corticotropin-releasing hormone, adrenaline, noradrenaline, and ghrelin are normally measured in such studies as stress indicators; less often stressinduced analgesia, behavioral characteristics are also taken into account

The restriction model of stress is one of the most popular (Gao et al., 2016). Sometimes the experimental animal is fixed with tapes, plaster, cloth towel, or other means so that only the head can move freely; however, most often for this purpose animal is settled in a plastic or metal tube, or a special microcell restricting its movements (Zhang et al., 2011; Gao et al., 2016). The duration of the procedure and the number of restriction episodes affect the intensity of the stress response and should be taken into account (Zhang et al., 2011; Wu X.F. et al., 2015; Zhao X.Y. et al., 2020).

Predator model of psychogenic stress is also widely used, for this purpose the natural predators of mice such as cats, ferrets, rats or foxes are normally chosen; sometimes not the predator itself, but its smell is offered to the tested mouse, this causes the fear and anxiety in the experimental animal (Sanchez-Gonzalez et al., 2018; Di Natale et al., 2019). The most commonly used version of the predator stress model for mice is the presentation of a hungry cat or its scent without physical contact between the mouse and the cat (Liu et al., 2012). The presence of the cat affects the stressed animal, and activating its hypothalamic-pituitary-adrenal axis, therefore triggering the secretion of glucocorticoids (Sanchez-Gonzalez et al., 2018).

Another widely used model is CUMS. Rodents are presented with constantly changing variable stressors over several weeks (Campos et al., 2013). According to this model, combination of isolation and overcrowding can be used as stressors, as well as unpredictable changing the situation in the cage: wet sawdust, tilting the cage, disruption of the day-night cycle, exposure to different temperatures, the use of mobility restrictions, social stress (Haller et al., 1999; Gao et al., 2016; Burstein, Doron, 2018; Gadek-Michalska et al., 2019). After several days of CUMS regimen, animals show an increase in blood corticosterone levels and a reduced response to pleasurable stimuli (Campos et al., 2013; Gadek-Michalska et al., 2019).

## Influence of stress on the reproductive function of mammals: experimental data

Animal studies demonstrate that psycho-emotional stress experienced by the female affects the quantity and quality of oocytes, which in turn contributes to further embryonic development (see the Table). Many studies come to the conclusion that stress leads to a decrease in the developmental potential of oocytes (Wiebold et al., 1986; Zhang et al., 2011; Liu et al., 2012; Lian et al., 2013; Wu X.F. et al., 2015; Gao et al., 2016); this, in turn, resulted in a reduced percentage of blastocysts developed from such oocytes. A decrease in the developmental potential of oocytes was associated with the duration and severity of the applied stress treatment (Gao et al., 2016). It was also revealed, that antral follicles are more sensitive to stress than preantral ones (Gao et al., 2016). Moreover, chronic unpredictable stress disrupts ovulation and cyclicity in female mice, these changes in reproductive system correlate with high levels of corticosteroids in the blood and with the increased activity of superoxide dismutase; moreover, after hormonally induced stimulation of superovulation, mature oocytes were not found in the stressed female mice (Kala, Nivsarkar, 2016).

**Table 1.**
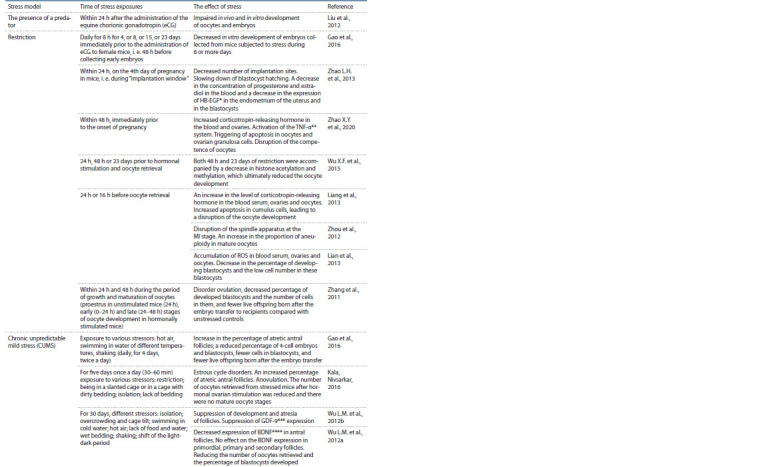
Effects of stress exposures on the development of oocytes and embryos in mice * HB-EGF, heparin-binding EGF-like growth factor. ** TNF-α, tumor necrosis factor alpha. *** GDF-9, growth differentiation factor 9. **** BDNF, brain-derived neurotrophic factor.

Stress can also affect embryo implantation. It was shown that even a short restriction stress lasting 24 hours, but coinciding in time with the “implantation window” on the fourth day after mating, negatively affects implantation in mice and slows down the onset of hatching in blastocysts (Zhao L.H. et al., 2013). This effect was mediated through a decrease in the blood levels of progesterone and estradiol, and was associated with the level of expression of heparinbinding epidermal growth factor both in the uterus and in the blastocysts (Zhao L.H. et al., 2013).

It is known that stress leads to activation of the hypothalamic- pituitary-adrenal and sympathoadrenal systems; therefore, traditional markers of stress are glucocorticoids and adrenaline. Restriction stress in mice was shown to be accompanied by an increase in plasma cortisol levels (Zhang et al., 2011). Cortisol injections also led to suppression in oocyte development. In addition, stress led to a decrease in the follicle-stimulating hormone (FSH) release, while injections of cortisol did not cause this effect. The researchers concluded that cortisol affects oocytes through a direct effect on the ovary, while stress impairs their competence indirectly, via effects on the hypothalamicpituitary- adrenal and hypothalamic-pituitary-ovarian axes (Zhang et al., 2011).

One of the ways to implement stressful effects on the female reproductive system is to influence the production of ovarian regulators of folliculogenesis, including those mediated by corticotropin-releasing hormone. Corticotropinreleasing hormone (CRH), which is identified in the theca and stroma of the ovaries, as well as in the cytoplasm of oocytes and granulosa cells, is involved in the regulation of follicular maturation, ovulation, the formation of corpus luteum, and the synthesis of the ovarian steroid hormones (Kiapekou et al., 2010; Zhai et al., 2020).

In female mice, restriction stress caused an increase of CRH concentration in blood serum, ovaries, and oocytes, as well as an increase in the expression of the CRH receptor 1 (CRHR1) in granulosa and theca cells, but a decrease in the expression of the glucocorticoid receptor and brainderived neurotrophic factor (BDNF) in the ovaries (Liang et al., 2013). All this ultimately led to an imbalance between estradiol and progesterone concentration in blood and negatively affected the developmental competence of oocytes. Besides, the addition of CRH to the culture medium during oocyte’s in vitro maturation disrupted its development and increased the rate of apoptosis in granulosa cells (Liang et al., 2013). In another study, it was shown that a stressinduced increase of CRH both in blood and in the ovaries of female mice triggers apoptosis in oocytes and in ovarian granulosa cells due to the activation of the TNF-α system, which results in impaired oocyte competence (Zhao X.Y. et al., 2020).

Animal experiments using CUMS stress model demonstrated that inhibition of follicle development is associated not only with gonadotropins, but also with growth factors such as growth differentiation factor 9 (GDF-9) and BDNF (Wu L.M. et al., 2012b). Exposure of female mice to CUMS resulted in the suppressed follicular development, increased level of follicular atresia, and downregulated GDF-9 expression. The introduction of exogenous gonadotropins partially mitigated these negative effects and restored the development of antral follicles, which was suppressed due to chronic stress, but these exogenous gonadotropins exerted no effects on secondary follicles. However, the introduction of recombinant GDF-9 restored the development of secondary follicles. Co-administration of GDF-9 and gonadotropins in stressed mice restored both secondary and antral follicles. Another study of the same research group showed that CUMS reduces BDNF expression in antral follicles but does not affect BDNF expression in primordial, primary, and secondary follicles (Wu L.M. et al., 2012a). Chronic unpredictable mild stress also reduced the number of retrieved oocytes and the percentage of blastocysts formed, which was corrected by the use of exogenous BDNF

Some studies attempt to elucidate mechanisms of the influence of psycho-emotional stress on the developmental potential of oocytes and preimplantation embryos. One study reported, that the transition of the heterochromatin configuration from the non-surrounded nucleolus (NSN) type to the surrounded nucleolus (SN) type is suppressed at the germinal vesicle (GV) stage preovulatory oocytes exposed to restriction stress, thus, the developmental potential of such oocytes is impaired (Wu X.F. et al., 2015).

Besides, psycho-emotional stress can lead to disruption of meiotic division in oocytes. It was shown that in stressed females, the proportion of aneuploidy in mature oocytes increases, and the percentage of aneuploid oocytes was three times higher in oocytes with accelerated maturation compared to the delayed ones (Zhou et al., 2012).The authors concluded that maternal stress may cause oxidative stress within oocytes and impair spindle assembly by inactivating the spindle-assembly checkpoint (Zhou et al., 2012).

In addition to hormonal imbalance, psychosocial stress causes an increase in the formation of reactive oxygen species (ROS). High levels of ROS cause oxidative stress, which leads to meiotic cell cycle arrest and resulted in apoptosis (Prasad et al., 2016; Chaudhary et al., 2019). This conclusion was further supported by the observation that oxidative stress induces granulosa cell apoptosis and leads to a decrease in estradiol levels, ovulation frequency, and oocyte quality (Tripathi et al., 2013). Besides, oxidative stress-induced apoptosis of granulosa cells caused the impairments of the contacts of these cells with oocytes, which directly affects the supply of nutrients and the availability of growth factors that affect the quality of oocytes in preovulatory ovarian follicles (Prasad et al., 2016). In experiments using the restriction stress model, it was shown that stress caused the accumulation of ROS in the blood serum of mice, ovaries, and oocytes, and also caused a decrease in the percentage of blastocysts developing in vitro with fewer cells observed in these blastocysts (Lian et al., 2013).

It should be noted that the mechanism of the negative impact of chronic stress on the ovary through inhibition of the release of gonadotropins has been well studied. While other ovarian regulatory mechanisms involved in this process are not yet understood. Elucidating these paracrine mechanisms mediating the effects of stress on oogenesis and, subsequently, on the development of embryos is important for more effective use of medical reproductive technologies in patients experiencing chronic psycho-emotional stress.

## Impact of stress on female reproductive function: clinical data

Clinical data without ART

Functional hypothalamic amenorrhea which may be diagnosed in the absence of menstruation for three or more months represents the most striking clinical manifestation of stress-induced disorders of folliculogenesis, with initially intact menstrual function (Warren, Fried, 2001). First of all, functional hypothalamic amenorrhea is characterized by a decrease in the frequency and amplitude of gonadotropinreleasing hormone release peaks, what leads to a decrease in the production of FSH and luteinizing hormone (LH) by the pituitary gland and, as a result, to the absence of hormonedependent follicle growth. This leads to a disruption of the transition from secondary follicles to antral follicles, a disruption of the formation of a pool of growing follicles, the absence of a dominant follicle and, accordingly, lack of the corpus luteum. Therefore, functional hypothalamic amenorrhea is characterized by a decrease in the production of estradiol in the ovaries, which is accompanied by the absence of uterine endometrial proliferation and the absence of menstruation (Fourman, Fazeli, 2015; Prokai, Berga, 2016).

Manifestations of other disorders of folliculogenesis caused by stress may not be as pronounced as amenorrhea. Subclinical manifestations include lengthening and irregularity of the menstrual cycle, insufficiency of the luteal phase of the cycle, luteinization of the non-ovulated follicles (Berga, Loucks, 2007; Palm-Fischbacher, Ehlert, 2014), as well as the absence of a mature oocytes in the ovulated follicle (Tamura et al., 2013). In the case of subclinical manifestations, there is also a decrease in the production of gonadotropin-releasing hormone, however, FSH still reaches a level sufficient to initiate follicle growth. In this case, the growth of the follicles may be slow, with the prolongation of the first phase of the cycle, the late ovulation, and the reduced production of estradiol, which can lead to endometrial hypoplasia (Berga, Loucks, 2007; McEwen et al., 2012). Changes in gonadotropin-releasing hormone pulsation also affect the intensity and frequency of LH peaks, although serum LH levels may remain normal or be only slightly reduced (Krsmanovic et al., 2009). Deficiency in the production of gonadotropin-releasing hormone and estradiol can lead to a reduced LH peak in the preovulatory period and to luteinization of the unovulated follicle, the formation of ovarian cysts, luteal phase insufficiency, and the reduced progesterone production in the second phase of the cycle (Berga, Loucks, 2007). Besides, chronic stress is characterized by increased secretion of cortisol mainly at night, with normal levels of daily and morning secretion (McEwen, 2000), which also contributes to a decrease in the amplitude or even the absence of the ovulatory peak of gonadotropins (Cahill et al., 1998).

Certainly, not all women demonstrate disruption of folliculogenesis and the distortion of the menstrual cycle in the stressful situations (McComb et al., 2006; Ellison et al., 2007). There are both physiological and psychological factors determining tolerance to stress-induced ovarian dysfunction (Wingfield, Sapolsky, 2003; Palm-Fischbacher, Ehlert, 2014). Factors that protect the reproductive system in the situation of stress operate at the level of the central nervous system (the stressor is not perceived), at the level of the hypothalamic-pituitary-adrenal system (impaired secretion of glucocorticoids), at the level of the hypothalamicpituitary- gonadal system (resistance of the gonads to the action of glucocorticoids), and protection from the action of glucocorticosteroids with the help of a proteins that bind steroids (Wingfield, Sapolsky, 2003).

The development of hypothalamic amenorrhea leads to the reproductive failure due to the absence of follicular growth and the lack of matured oocytes, as well as the lack of appropriate preparation of the endometrium. Sometimes in the case of stressful influences with the formation of luteinization of the unovulated follicle and a deficiency of the luteal phase, the absence of menstruation (amenorrhea) is not observed, but the reproductive potential is significantly reduced (Lynch et al., 2014). Such alterations can be corrected by the proscription of appropriate medical treatment that compensate the deficiency of pituitary and/or steroid hormones. However, there is the problem of infertility of unknown origin, with unimpaired folliculogenesis and ovulation. A more detailed study of oogenesis became possible with the introduction of ART.

There are several evidences of a high rate of early pregnancy loss, not associated with chromosomal abnormalities, or an increased anxiety and depression in women with a history of miscarriages. Pregnancy loss itself may be considered as a powerful stressor, which can lead to the recurrent miscarriage (Quenby et al., 2021; Wang et al., 2021). Early pregnancy loss indicates a low viability of the embryo or lack of its interaction with the uterus after implantation, which is probably the result of gametogenesis disruption, distortion in preparation of the endometrium for pregnancy or the development of immunological incompatibility between the maternal organism and the embryo. All of these conditions have been described as possible consequences of stress experienced by a woman during conception and early pregnancy (Wadhwa, 2005; Nepomnaschy et al., 2006). These phenomena can lead to impaired placentation and the development of pregnancy complications typical for later stages of gestation, such as fetoplacental insufficiency, preeclampsia, preterm birth; these conditions may affect the health of children born (Parker, Douglas, 2010; Witt et al., 2012). Mechanisms which cause a change in ovarion function under stress are shown in the Figure.

**Fig. 1.**
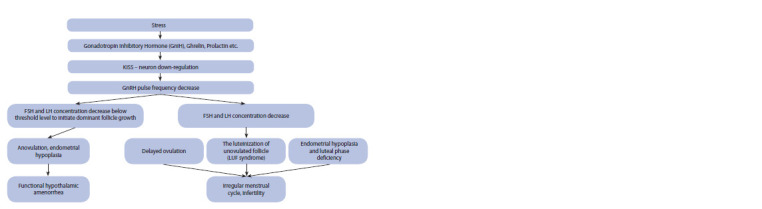
Effects of stress on female reproductive function.

## Clinical data obtained with the use of ART

The use of gonadotropins to induce the growth of several follicles for controlled ovarian hyperstimulation is one of the core ARTs. Thus the stress-induced deficiency of CRH, FSH, and LH, which was discussed in the previous section, is compensated by the administration of exogenous gonadotropins. Moreover, current protocols for controlled ovarian hyperstimulation involve blockade of endogenous gonadotropin-releasing hormone production in order to prevent premature ovulation. Taking all this into account, it can be concluded that during the use of ART, stress-induced disturbance of oogenesis is realized not through a decrease in the production of hormones of the hypothalamus and the pituitary gland, but via other paracrine and autocrine mechanisms described above.

Many reproductologists noticed that unsuccessful ART attempts pretty often take place during adverse life events, such as death of a relative, family problems, etc. This is in practice described by reproductologists as cases of “inexplicably low” quality/quantity of oocytes and embryos in ART programs with an initially good prognosis, and “unexplained” improvement in the quality/quantity of oocytes and embryos in repeated attempts using the same protocols when life situation of the patient was improved (Ebbesen et al., 2009; Meldrum, 2016). A possible explanation for such observations is the inhibitory effect of the stress on gametogenesis.

Data from clinical studies in humans are contradictory. A significant part of these studies indicates the depressing effect of psychological stress on the results of ART. Thus, in a study by Ebessen et al. (2009) involving 809 women practicing ART for the first time, a decrease in the number of received oocytes, embryo quality and pregnancy rate was shown with an increase in the number of adverse life events that reduce the quality of life, as well as with an increase in the level of perceived stress one month before infertility treatment with ART (Ebbesen et al., 2009). Another study showed the effect of initial stress on the number of oocytes retrieved and fertilized, as well as on pregnancy and live birth rates (Klonoff-Cohen et al., 2001). Li et al. (2011) found that initial psychological stress was negatively associated with pregnancy rates in ART programs, but intrafollicular concentrations of norepinephrine did not differ between the pregnant and non-pregnant women. In another study, the association of ART results with anxiety levels and serum concentrations of cortisol and noradrenaline was investigated. Elevated levels of cortisol and norepinephrine have been shown to be associated with anxiety levels and negatively correlated with pregnancy and live birth rates (An et al., 2013).

A more recent paper published the results of a study with 135 women involved, that examined the association of salivary and hair cortisol with ART outcomes (Massey et al., 2016). It was shown that salivary cortisol levels were not predictive of ART outcomes. Whereas, lower hair cortisol concentrations predicted the high probability of pregnancy. A recent study of 304 women found that more than 80 % of respondents had elevated levels of anxiety and depression, and these symptoms were inversely correlated with the success of ART implementation (Aimagambetova et al., 2020).

At the same time, some studies do not show an association between anxiety levels, as well as salivary and serum cortisol levels and reproductive outcomes in ART patients (Lovely et al., 2003; Cesta et al., 2018). Miller et al. (2019) assessed the level of anxiety using the Perceived Stress Scale, salivary cortisol concentration at the beginning of the ART cycle, on the day of follicle puncture, and on the day of embryo transfer, and also measured the level of cortisol in the follicular fluid. The authors noted an increase in cortisol and anxiety on the day of follicle puncture, but did not find an association of these stress indicators with pregnancy rates. Besides, elevated follicular cortisol levels correlated with positive ART outcomes. In vitro fertilization (IVF) failure has also been shown to predict subsequent psychological distress, but pre-IVF psychological distress did not predict IVF failure (Pasch et al., 2012). The level of stress and the number of oocytes obtained in ART programs for the treatment of infertility was compared. The results of this study showed a significantly higher level of stress in patients with infertility, but the number of oocytes was comparable in both groups (Adeleye et al., 2020).

It can be concluded that despite the large number of publications addressing the effects of stress on the effectiveness of ART in humans, the data obtained are very contradictory. In these studies not only sizes of study groups are variable, but also different approaches to assess experienced stress and anxiety were used. Moreover, many of these studies suffer from the lack of randomization. The majority of these studies do not take into account the fact that the patient’s knowledge of his prognosis can significantly affect the assessment of the level of chronic stress and the results of the questionnaire. Due to the heterogeneity of the published data, the conclusions of these works are also contradictory. Most authors are careful in conclusions about the relationship between stress and reproductive function, based both on the data of their studies and on the general biological considerations suggesting the impossibility of complete suppression of the reproductive function during unfavorable periods due to the need for the survival of the species (Wingfield, Sapolsky, 2003; Rooney, Domar, 2018; Lawson, 2020).

## Psychotherapy as a way to mitigate the negative effect of psycho-emotional stress on the reproductive system

The availability of data indicating a significant impact of psychosocial stress on the reproductive system has contributed to an increase in research aimed at studying psychotherapeutic effects in the treatment of infertility.

An early paper addressing this issue highlights the urgent need for quality-compliant research feasible for evaluation (Boivin, 2003). The author analyzed 38 studies, 25 of which were classified as independent, and only eight of them met the research quality standards. In summary, three out of eight good quality studies showed higher pregnancy rates in the psychosocial intervention group compared to the routine care group (Boivin, 2003). In another paper, a metaanalysis of 22 studies was conducted, which indicates that psychotherapy (group and individual/couple) reduced anxiety and depression in infertile patients and possibly affected the success rate of conception (Liz, Strauss, 2005). A review by Campagne (2006) recommends planning of infertility medication taking into account the level of stress, and suggests stress-reducing therapies, prior to initiating infertility treatment (Campagne, 2006).

Subsequent studies presented conflicting results of the use of psychological techniques. Hämmerli et al. (2009) included 21 controlled trials in their meta-analysis and concluded that psychological interventions were not associated with any significant changes in psychological status, but had a positive effect on pregnancy rates in patients receiving treatment without ART (Hämmerli et al., 2009). They also concluded that a therapy of six or more sessions was more effective than a shorter duration of therapy. Frederiksen et al. (2015) performed a meta-analysis of 39 original articles and reported that women receiving some form of psychotherapeutic intervention were about twice as likely to become pregnant compared with women receiving standard treatment (Frederiksen et al., 2015).

Ying et al. (2016) included 20 randomized trials in their systematic review. They concluded that there were methodological problems with studies that reported significant effects of psychological stress on the pregnancy rates, and recommended that a more thorough investigation to be conducted, especially for the most stressful period for infertile patients, in particular, during the time of waiting for the results of a pregnancy test. In a systematic review by Gaitzsch et al. (2020), only two of six studies showed a significant positive effect of psychological interventions on the fertility (Gaitzsch et al., 2020). At the same time, a meta-analysis including 15 studies showed a positive association between psychosocial interventions, especially long-term ones, and pregnancy rates in infertile women and couples receiving ART treatment (Katyala et al., 2021).

Thus, many researchers emphasize the presence of methodological and practical questions to the currently accumulated data. There is a need for more studies and for unified programs of psychological help. The positive effects of psychotherapy demonstrated in some of the studies indicate that this is a promising area for further research.

## Conclusion

The identification of chronic psycho-emotional stress is challenging both in humans and in experimental studies with laboratory animals. The psychological tests and questionnaires in humans are considered as the “gold standard” for such psycho-emotional stress identifying, however, it requires a lot of time, may not reflect the real physiological situation due to subjective distortions introduced by the interviewee (Slavich, Shields, 2018; Crosswell, Lockwood, 2020). It is also important that the use of psychological tests and questionnaires is not possible in experiments with laboratory animals. Therefore, the search for reliable biomarkers of chronic psycho-emotional stress which can be objectively measured and evaluated is extremely important.

The use of animal models helps to understand the mechanisms underlying the impact of assisted reproductive technology accompanied by stress on the female reproductive function and on the offspring health. Analysis of the literature let to conclude that stress negatively affects the development of ovarian oocytes, as well as the subsequent embryo development. The role of some ovarian paracrine factors that are involved in these processes has been revealed in these studies. Meanwhile, additional experiments on the effect of psycho-emotional stress on the results of in vitro fertilization and embryo transfer experiments are warranted, since clinical data are contradictory and only a few experimental works on laboratory animals are available so far.

Available data on the laboratory animals show the effectiveness for the use of such factors as GDF-9 and BDNF to reduce the inhibitory effect of stress on folliculogenesis and embryo development, these factors are promising to be used in the reproductive medicine. Moreover, psychotherapeutic techniques which alleviate effects of stress may increase resistance to stress at the level of the central nervous system, i. e. influence the perception of a stressful event or stimulus. There are reports confirming the effectiveness of psychological techniques in reducing psychological stress, and there are evidences that the use of these techniques is associated with a significant increase in pregnancy rates (Hämmerli et al., 2009; Frederiksen et al., 2015; Katyala et al., 2021). It is important to increase the availability of psychotherapy in reproductive medicine, especially taking into account the level of stress reported by infertile women.

## Conflict of interest

The authors declare no conflict of interest.
